# Non-Invasive Glucose Measurement by Use of Metabolic Heat Conformation Method

**DOI:** 10.3390/s8053335

**Published:** 2008-05-21

**Authors:** Fei Tang, Xiaohao Wang, Dongsheng Wang, Junfeng Li

**Affiliations:** State Key Laboratory of Precision Measurement Technology and Instruments, Department of Precision Instruments and Mechanology, Tsinghua University, Beijing 100084, People's Republic of China

**Keywords:** sensor, heat dissipation, blood flow rate, degree of blood oxygen saturation, evaporation

## Abstract

A non-invasive glucose measurement system based on the method of metabolic heat conformation (MHC) is presented in this paper. This system consists of three temperature sensors, two humidity sensors, an infrared sensor and an optical measurement device. The glucose level can be deduced from the quantity of heat dissipation, blood flow rate of local tissue and degree of blood oxygen saturation. The methodology of the data process and the measurement error are also analyzed. The system is applied in a primary clinical test. Compared with the results of a commercial automated chemistry analyzer, the correlation coefficient of the collected data from the system is 0.856. Result shows that the correlation coefficient improves when the factor of heat dissipated by evaporation of the skin is added in. A non-invasive method of measuring the blood flow rate of local tissue by heat transmission between skin and contacted conductor is also introduced. Theoretical derivation and numerical simulation are completed as well. The so-called normalized difference mean (NDM) is chosen to express the quantity of the blood flow rate. The correlation coefficient between the blood flow rates by this method and the results of a Doppler blood flow meter is equal to 0.914.

## 1. Introduction

Diabetes is a common disease related to endocrine metabolism. At present there is no method which can cure diabetes totally. The main therapy is to prevent or alleviate the occurrence of complications through frequent monitoring and adjustment of the glucose level. Physicians suggest that the glucose level should be tested at least four times per day. A non-invasive method could realize this requirement easily, because it is painless and does not infect the patient with other diseases. If it works, it would be the most effective way to make the diabetic's life easier.

At present, the research on non-invasive glucose measurement focuses mainly on the technique of spectrum detection, such as near infrared detection [1-4], mid-IR detection [5], Raman Spectrum detection [6], optoacoustic detection [7], polarimetry [8], and so on. Though these methods have been studied for years, they have remarkably achieved no great improvements in sensitivity and accuracy. In this paper, a non-invasive glucose measurement system based on the MHC method is developed and the clinical tests show that this method has good effect and high optimization potential.

## 2. Detection Principle

The homoeostatic circadian rhythm of the human body depends on the correlation between metabolic heat, local oxygen supply level and glucose level. Glucose and oxygen are supplied to the cells in the body through the blood circulation system. The oxidation of glucose is related to the generation of energy which can be emitted into the environment in the form of heat, so the quantity of dissipated heat is correlated to the quantity of dissipative glucose and oxygen [9]. The MHC method is based on this. Since the quantity of supplied oxygen is the function of the degree of blood oxygen saturation and the blood flow rate in the capillary vessel, the quantity of dissipated heat will be

(1) H=f(G,BF,O)

where *H* is the quantity of dissipated heat, *G* is the glucose level, *BF* is the blood flow rate and *O* is the degree of blood oxygen saturation. The glucose level can be obtained as long as *H*, *BF* and *O* are measured.

The main forms of the heat emitted in the environment are radiation, convection and evaporation. The heat dissipated through radiation is related to the skin's surface temperature and ambient temperature. According to the Stefan-Boltzmann law, the heat transferred by radiation can be obtained through measurement of the skin's surface temperature by the infrared sensor and measurement of the ambient temperature by the thermal resistor, which is

(2) $$R=\delta \cdot S\;(T_{S}^{4}-T_{A}^{4})$$

where *R* is the quantity of heat dissipated by radiation, *δ* is the coefficient of radiation, *S* is the area of radiation, *T_S_* is the absolute temperature of the surface and *T_A_* is the absolute temperature of ambience.

The heat dissipated by convection is also conditionally related to the surface temperature and ambient temperature. According to Newton's formula of cooling, the transferred heat is

(3) $$C=h_{c}\;(T_{S}-T_{A})$$

where *C* is the quantity of heat transferred by convection and *h_c_* is the coefficient of heat transferred by convection.

The heat dissipated by evaporation is related to the quantity of evaporation from the skin's surface. The skin is supposed to be dry and there is no sweat to make the skin wet. Then the skin is in the state of insensible perspiration which Vanger thinks is not controlled by the regulating system of heat, and can be expressed as

(4) $$E=r\cdot m\left(p_{sk}-p_{a}\right)$$

where *E* is the heat dissipated by evaporation, *r* is the latent heat of vaporization, *m* is the permeability coefficient of the skin, *p_sk_* is saturated pressure component of the water vapour in the air on the skin's surface and *p_a_* is partial pressure of ambient water vapour.

When the skin surface contacts a heat conductor with lower temperature, heat transmission will occur between them. Then the surface temperature changes, and so does the temperature of the heat conductor's two ends. The quantity of variation relies on the quantity of heat transferred from the surface to the heat conductor, which relies on the blood flow rate in the capillary vessel. So the blood flow rate can be calculated through measurement of the temperature change of the conductor's ends. This method of measurement will be described in the next section.

The degree of blood oxygen saturation can be measured by the optical method, for which the formula is

(5) $$\frac{c_{\text{hbo}2}}{c_{\text{hbo}2}+c_{hb}}\times 100\%=\frac{k_{2hb}\Delta A_{1}-k_{1hb}\Delta A_{2}}{k_{2hb}\Delta A_{1}+k_{1\text{hbo}2}\Delta A_{2}-k_{2\text{hbo}2}\Delta A_{1}-k_{1hb}\Delta A_{2}}\times 100\%$$

where *C_hbo_*_2_ and *C_hb_* are concentrations of oxyhaemoglobin and haemoglobin respectively, *k*_1_*_hb_* and *k*_2_*_hb_* are extinction coefficients of haemoglobin to two kinds of light with different wavelengths, *k*_1_*_hbo_*_2_ and *k*_2_*_hbo_*_2_ are extinction coefficients of oxyhaemoglobin to two kinds of light with different wavelengths and Δ*A*_1_ and Δ*A*_2_ are the intensities of two kinds of light with different wavelengths absorbed by arterial blood.

## 3. Principle and Simulation of Blood Flow Rate Measurement by Use of Thermal Diffusion

**Figure 1.**
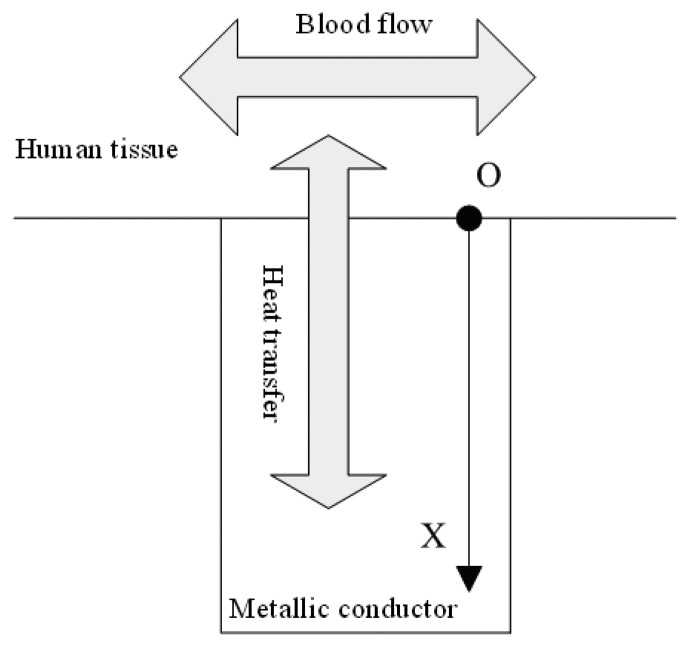
Blood flow rate measurement by use of thermal diffusion.

When the skin contacts a metallic conductor of lower temperature, heat transmission will occur between them, and the temperature of the skin will change, as shown in Fig. 1.

After the variation of the dermal temperature, heat transmission will occur between the blood and skin for the temperature difference. The quantity of heat transferred from blood to skin is

(6) $$\text{d}w_{1}=\left(T_{2}-T_{1}\right)\times c_{1}\times \rho_{1}\times v_{1}\times \text{d}t$$

where *T*_2_ is the skin temperature, *T*_1_ is the blood initial temperature, *c*_1_ is the heat capacity of the blood, *ρ*_1_ is the density of the blood and *v*_1_ is the flow rate of the blood.

In the time of d*t*, the quantity of heat transmitted from the contact point of the finger to the conductor is

(7) $$\text{d}w_{2}=\left[T_{2}-T_{3}\left(0\right)\right]\times u_{2}\times \text{d}t$$

where *T*_3_(0) is the temperature of the conductor at the contact point and *u*_2_ is the heat transfer coefficient between the skin and the conductor.

In the conductor, the quantity of heat transmitted is

(8) $$\text{d}w_{3+}\left(x\right)={T\prime }_{3+}\left(x\right)u_{1}\times s\times \text{d}t$$

(9) $$\text{d}w_{3-}\left(x\right)={T\prime }_{3-}\left(x\right)u_{1}\times s\times \text{d}t$$

where d*w*_3+_ is the quantity of heat transmitted from any point in the conductor to the positive x axis, d*w*_3-_ is the quantity of heat transmitted from any point in the conductor to the negative x axis, *T*′_3+_ is the right-hand derivative of the temperature, *T*′_3-_ is the left-hand derivative of the temperature, *u*_1_ is the coefficient of heat conduction of the conductor and *s* is the cross-sectional area of the conductor.

The quantity of heat change in the skin of the finger is the difference between the quantity of heat transmitted from blood to the skin and the quantity of heat transmitted from the skin to the conductor

(10) $$\text{d}T_{2}\times m_{2}\times c_{2}=\text{d}w_{1}-\text{d}w_{2}$$

where *m*_2_ is the equivalent mass of the finger skin and *c*_2_ is the equivalent heat capacity of the finger skin.

The equation of temperature change at the contact point between the conductor and the skin is

(11) $$\text{d}T_{3}\left(0\right)\times s\times \rho_{3}\times c_{3}\times \text{d}x=\text{d}w_{2}-\text{d}w_{3+}\left(0\right)$$

where *ρ*_3_ is the density of the conductor and *c*_3_ is the heat capacity of the conductor.

The equation of temperature change at any point in the conductor is

(12) $$\text{d}T_{3}\left(x\right)\times s\times \rho_{3}\times c_{3}\times \text{d}x=\text{d}w_{3-}\left(x\right)-\text{d}w_{3+}\left(x\right)$$

The equation of temperature change at the other end of the conductor is

(13) $$\text{d}T_{3}\left(L\right)\times s\times \rho_{3}\times c_{3}\times \text{d}x=\text{d}w_{3-}\left(L\right)$$

where *L* is the length of the conductor.

The temperature variation curve at any point of the conductor with different blood flow rate can be obtained from (6) - (13) by programming. The temperature change at any point of the conductor can reflect the changes of blood flow rate theoretically, so there are two methods to express the blood flow rate, which are the temperature change at the near end of the conductor to the skin and the temperature difference between the near end and the far end.

To compare the temperature curve at different skin temperatures and ambient temperatures, the temperature at any point of the conductor is normalized. The formula is

(14) $$T\left(t\right)=\left(T_{\text{x}}\left(t\right)-T_{c}\right)/\left(T_{\text{s}}-T_{c}\right)$$

where *T*(*t*) is the normalized temperature, *T*_x_(*t*) is the temperature at any point of the conductor, *T_s_* is the initial of the skin and *T_c_* is the initial temperature of the conductor.

**Figure 2.**
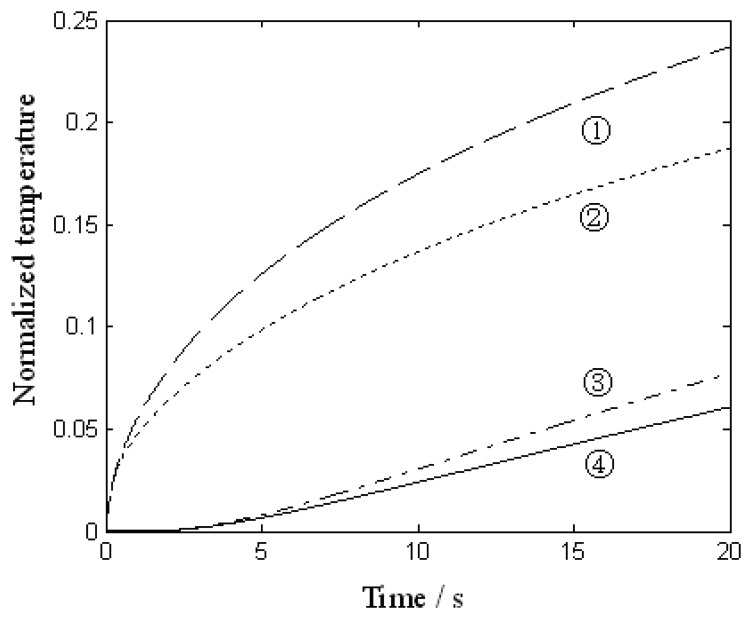
The normalized temperature curve at the two ends of the metallic conductor.

With the initial temperature of blood at 33 degrees Celsius, skin at 33 degrees Celsius and metallic conductor at 23 degrees Celsius, the normalized temperature curves at the contact point between the conductor and the skin, and the curves at the other end of the conductor at blood flow rate *ν*_b1_ and *ν*_b2_, *ν*_b1_< *ν*_b2_, are shown in Fig. 2. Lines 1 and 3 are the curve at the contact point and the curve at the other end at *ν*_b2_. Lines 2 and 4 are the curve at the contact point and the curve at the other end at *ν*_b1_. It can be concluded from the figure that when the blood flow rate is larger, the rate of normalized temperature rise is larger.

**Figure 3.**
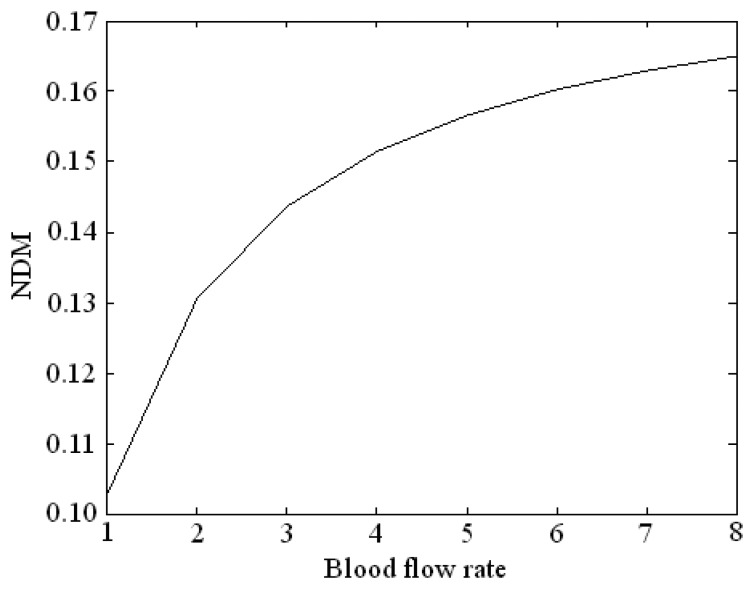
Relationship of the blood flow rate and the NDM

The normalized temperature curve can be reflected quantitatively by median value, slope or mean. Since the median value and the slope use only several points of the curve, local error has greater influence on the result. Thus the so-called normalized difference mean (NDM) is chosen to express the magnitude of the blood flow rate. The NDM is defined as the difference of the area integral of the two normalized temperature curves at the two ends of the conductor divided respectively by the integral length of the x axis at a blood flow rate. The NDMs for different blood flow rates, are shown in Fig. 3. It can be seen that when the blood flow rate is larger, the NDM is larger.

## 4. System Composition

**Figure 4.**
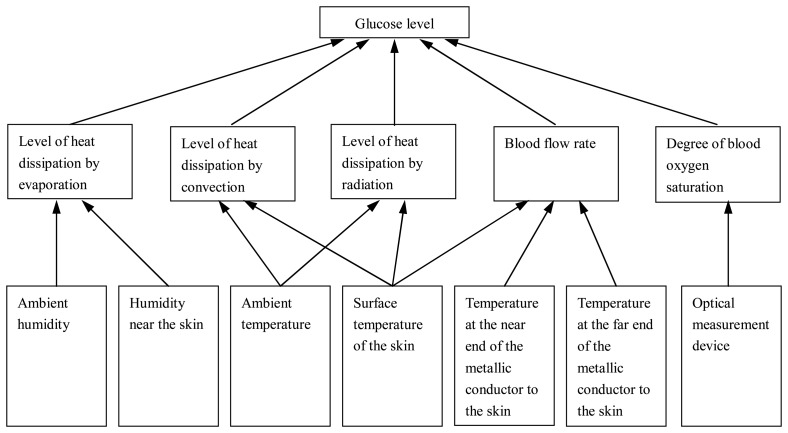
Schematic depiction of the detection system composition.

Through temperature sensors, a infrared sensor, humidity sensors and a optical measurement device, the detection system transforms information about temperature, humidity, blood flow rate and degree of blood oxygen saturation to find the glucose level, as shown in Fig. 4.

**Figure 5.**
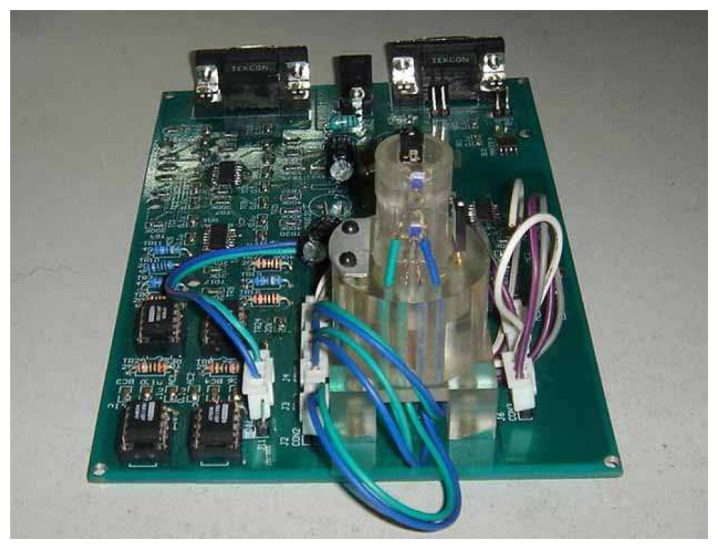
Photo of prototype of the non-invasive glucose measurement instrument.

Figure 5 is a photo of the prototype of non-invasive glucose measurement system based on the MHC method.

**Figure 6.**
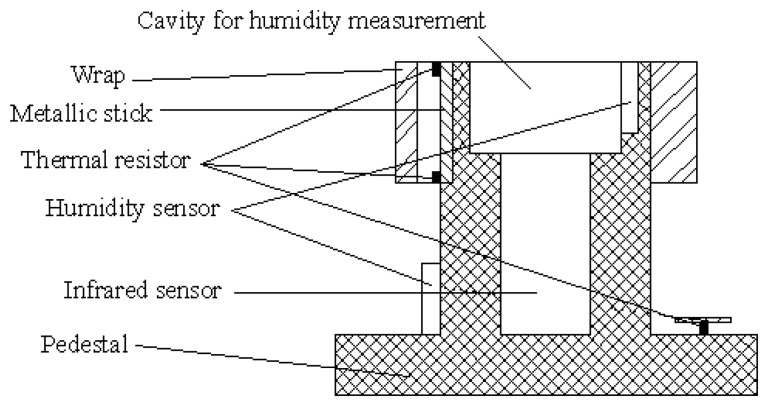
Structure of the detecting head.

The structure of the detecting head in the photo is shown in Fig.6. The degree of blood oxygen saturation is measured through an independent module.

## 5. Data Processing

According to the MHC theory, the glucose level can be estimated through the heat, blood flow rate and the degree of blood oxygen saturation produced by metabolism. In the normal range, their relation can be expressed as linear [9], which is

(15) $$G=a_{0}+a_{1}\times H+a_{2}\times BF+a_{3}\times O$$

The quantity of heat, estimated through radiation, convection and evaporation, can be divided into three parts, so it will be

(16) $$G=a_{0}+a_{1}\times R+a_{2}\times C+a_{3}\times E+a_{4}\times BF+a_{5}\times O$$

Parameters of *R*, *C*, *E*, *BF*, and *O* can be calculated by the data acquired from the sensors. After these parameters are normalized, the coefficient *a*_i_ in (16) can be determined by the method of partial least square regression [10]. In measurement, the glucose level can be estimated by the normalizing of the original data acquired and then by analysis with least square analysis.

## 6. Experiment and Discussion

### 6.1. Experiment: Blood Flow Rate Based on Heat Diffusion

The hardware used in the experiment to measure blood flow rate includes a detecting head, preamp filter circuit, AD converter and computer. Two thermal resistors, an infrared sensor, a pedestal and the metallic conductor comprise the detecting head. The thermal resistors are applied to measure the temperature at the two ends of the conductor. The infrared sensor is used to measure the skin's surface temperature. The pedestal is made of organic glass which has small heat capacity and low coefficient of heat conductivity, and can preserve the heat emanation and decrease the ambient influence on the measurement.

**Figure 7.**
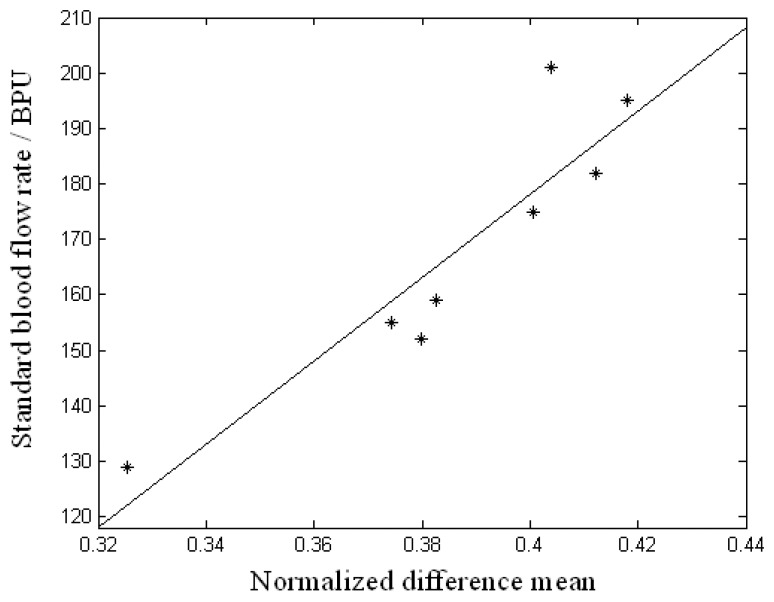
Result of the measurement of blood flow rate.

This system was applied in the measurement of volunteers. The measured values acquired by a Doppler blood flow meter of the physiological recorder of type MP150A-CE from company BIOPAC were set as the standard blood flow rates. In the test, to obtain different blood flow rates, volunteers were placed in several different situations: after 10 minutes sitting still, after 10 minutes standing, after 10 minutes standing and 30 push-ups, after 10 minutes standing and 30 deep squats, after 10 minutes standing and climbing 3 floors, after 10 minutes standing and climbing 10 floors, etc. Immediately after each exercise, the blood flow rate was measured in a specific area of his/her palm for about 10 seconds with the Doppler blood flow meter, and then with the experiment system sequentially. Fig. 7 shows the relation between the standard blood flow rate and the NDM measured by the system. The correlation coefficient is equal to 0.914.

### 6.2. Experiment: Non-invasive Glucose Measurement System

The clinical tests were made with the developed non-invasive glucose measurement system. Results of the first few tests were not good, with the correlation coefficient being no more than 0.4. The error mainly comes from the temperature difference between inside and outside the room, because the tests were completed in winter.

**Figure 8.**
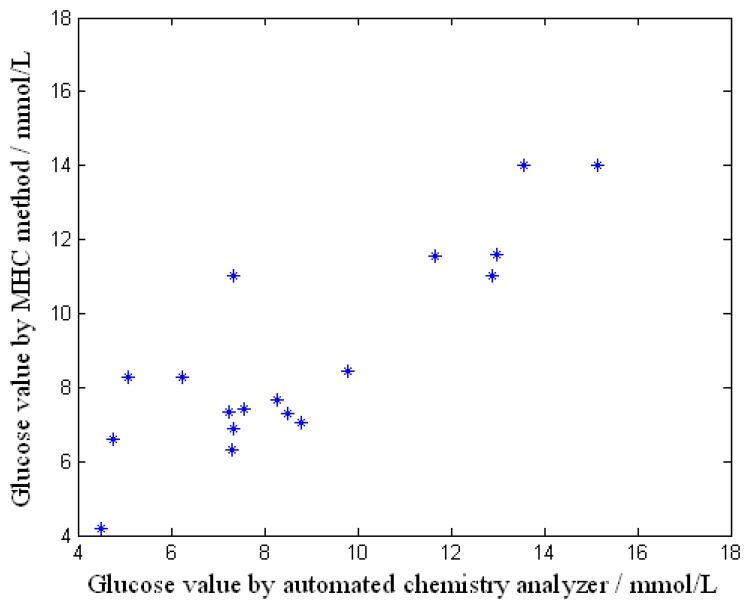
Result of the non-invasive glucose measurement.

Better results were obtained from the tests made in May when the temperature was good and the volunteers were in a neutral condition of heat. The volunteers in the tests were diabetics who were rechecking their clinical tests and rested for a moment inside the room. Fig. 8 shows the relationship of the glucose values obtained by the developed measurement system and the automated chemistry analyzer. The correlation coefficient is equal to 0.856. In this test, 18 effective fasting glucose samples were collected from 10 men and 8 women aged between 28 and 68 who had all been ill for 15 years. The temperature outside was 21-33 degrees Celsius and the temperature inside was 25-29 degrees Celsius.

**Figure 9.**
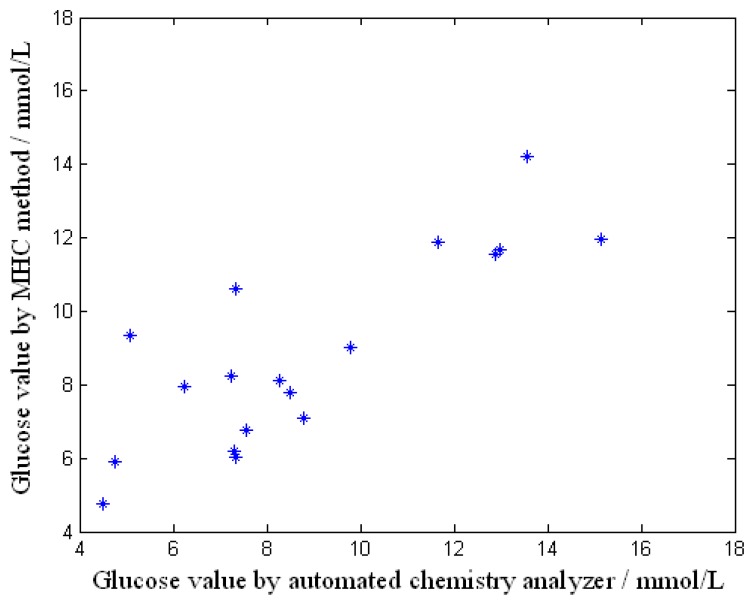
Result of the non-invasive glucose measurement without the influence of evaporation.

The relationship of the data from Fig.8 without the influence of evaporation is shown in Fig.9. The correlation coefficient is 0.823. It can be concluded that the correlation coefficient improves when the factor of heat dissipated by evaporation of the skin is added in.

A follow-up measurement with an individual cannot be done because of the constraint on resources. The various physical situation of each person results in the deviation of measured value among individuals, the accuracy is therefore influenced. If the difference between individuals could be eliminated, for example, the follow-up measurement uses the same individual, the accuracy of the measurement could be higher.

## 7. Conclusion

The paper discusses a non-invasive glucose measurement system based on the MHC method and the prototype is developed. Clinical tests were made on people who had been physically examined, both outpatients and clinical patients. The data collected were analysed. The correlation coefficient of the result of the system compared with a commercial automated chemistry analyzer is equal to 0.856. The method of measuring the blood flow rate with heat diffusion was devised, and numerical simulation and experimental verification carried out. The NDM was chosen to express the quantity of the blood flow rate. The correlation coefficient of the blood flow rate between this method and the Doppler blood flow meter is equal to 0.914. The paper also analyses the addition of the evaporation and shows that it can improve the measured result of the system.
